# Folate in autism neurodevelopment

**DOI:** 10.3389/fnut.2026.1927637

**Published:** 2026-08-19

**Authors:** George Ayoub

**Affiliations:** Psychology Department, Santa Barbara City College, Santa Barbara, CA, United States

**Keywords:** autism spectrum disorder, bioavailability, folate, folate receptor alpha autoantibodies, folic acid, folinic acid, leucovorin, neurodevelopment

## Abstract

Folate (vitamin B9) and vitamin B12 are essential micronutrients for cell growth, DNA synthesis, methylation, and one-carbon metabolism, with particular relevance to early development. This review begins with an overview of vitamins essential to development, with emphasis on folate and B12, followed by a detailed examination of folate sources, chemical forms, absorption, bioavailability, and genetic influences on folate metabolism and transport. We then address neurodevelopmental disorders, focusing on autism spectrum disorder (ASD), and synthesize evidence on folate’s role in brain formation during pregnancy, maternal folate deficiency as a contributor to autism risk, and critical periods during which folate status may most strongly influence neurodevelopmental outcomes. We focus specifically on clinical trial evidence: detection of folate deficiency or insufficiency in ASD children and treatment with leucovorin (folinic acid), as well as detection of folate deficiency risk in pregnancy, particularly through screening for folate receptor alpha autoantibodies (FRAA), and treatment with calcium folinate. Recent randomized and placebo-controlled trials suggest that reduced folate forms may offer benefits over folic acid in selected populations, including ASD children and FRAA-positive pregnancies, though larger confirmatory studies are needed. Taken together, this review highlights folate and B12 status as modifiable, biomarker-guided targets for supplementation strategies across the developmental life course, from preconception through childhood.

## 1 Vitamins in neurodevelopment

Healthy development depends on the integrity and function of endothelial, neuronal, and other cell populations whose growth and maturation are sensitive to micronutrient availability. Inadequate vitamin status during critical developmental windows, particularly in utero and in early childhood, can impair cellular processes including DNA synthesis, methylation, and antioxidant defense, with downstream consequences for vascular, neurological, and immune development. Vitamins, particularly B-group vitamins, as well as vitamins C, D, and E, play crucial roles in supporting these processes. This review introduces the vitamins most relevant to development, with a special emphasis on vitamin B9 (folate), its forms, bioavailability, and the consequences of deficiency or insufficiency of B9 and B12 for developmental outcomes.

The papers cited were procured first through weekly PubMed searches for folate and/or B12 AND development or autism spectrum disorder (ASD) or autism, which we have used for over 5 years. This was supplemented by *de novo* searches with the same terms in PubMed and Google Scholar to identify non-PubMed listed reports through May 2026. All reports identified with data regarding developmental processes in ASD or related neurodevelopmental disorders were included.

### 1.1 Vitamins essential for one-carbon metabolism: folate

Folate (Vitamin B9) is essential for one-carbon metabolism, which supports DNA and RNA synthesis, facilitating cell division and repair (1–3). Folate in one-carbon metabolism also supports methylation reactions, which regulate genes, as well as amino acid metabolism (2).

Vitamins B9 and B12 are essential to the conversion of homocysteine to methionine. Reducing homocysteine lowers its toxicity that can otherwise harm blood vessels (1, 4).

**Figure 1:**
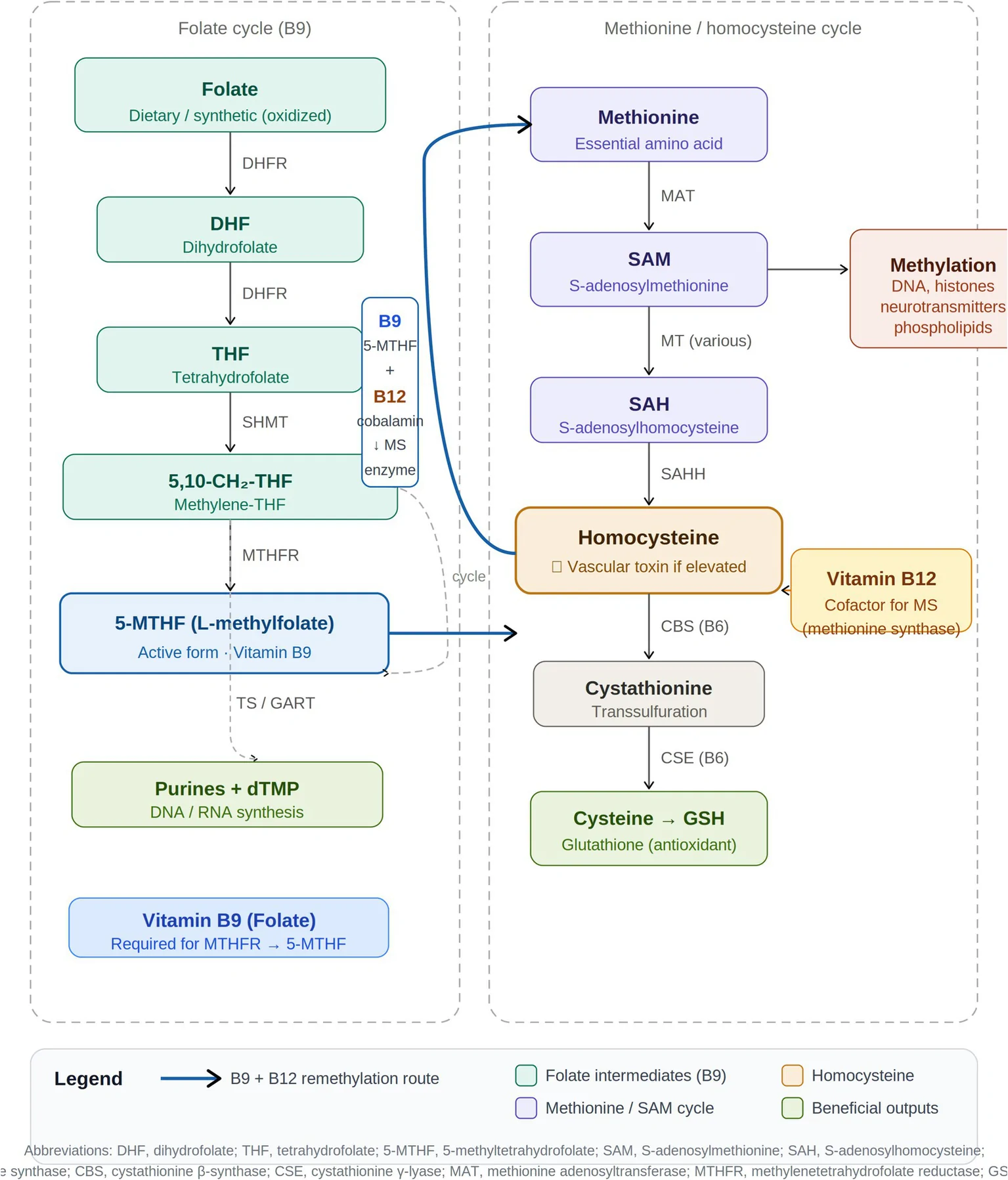
Metabolism of vitamin B-9. Left panel (Folate cycle/B9): Conversion sequence from dietary folate/folic acid → DHF → THF → 5,10-methylene-THF → 5-MTHF (L-methylfolate, the active form), with the MTHFR enzyme step labeled. A branch shows how folate intermediates also feed DNA/RNA synthesis (purines + dTMP). Right panel (Methionine / Homocysteine cycle): The full methionine cycle: Methionine → SAM → SAH → homocysteine (highlighted in amber as the key vascular toxin), with SAM’s role in methylation reactions (DNA, histones, neurotransmitters) branching off. Below homocysteine, the transsulfuration pathway (B6-dependent) leads to cystathionine → cysteine/glutathione. The connecting bridge: The thick blue arrow shows 5-MTHF + B12 (methionine synthase) remethylating homocysteine back to methionine: The critical step where B9 and B12 act together to keep homocysteine low (32, 81).

There are two forms of dietary folate. The natural form is found in foods (leafy greens, legumes, liver). It is reduced in form, and requires conversion in the body to the active form utilized by cells (1, 3). The synthetic, more stable form is oxidized, and is used in supplements and food fortification. It is better absorbed (~85% bioavailability) than food folate (~50%), but must first be reduced before it can then be converted to the active form 5-methyl-(6S)-tetrahydrofolate (L-5-MTHF), or L-methylfolate) in the liver and digestive tract (1, 3). Figure 1 shows the pathways of dietary folate and folic acid and their conversion to L-methylfolate, which is the biologically active form (4). While supplements typically contain the synthetic folic acid due to its long-term stability, some use L-methylfolate.

One variation in supplements is whether the active L-methylfolate is used or the racemic version, which includes the D-isomer in equal amounts. If the supplement is a racemic mix, then the dosing needed is twice that of the active L-methylfolate. This is true for L-methylfolate as well as for the other reduced form of folate, folinic acid (leucovorin), where the active form is levofolinic acid (levoleucovorin). L-methylfolate is relatively commonly available now, while leucovorin/levoleucovorin are used in approved drug products. In this paper, we use the term methylfolate to refer to the active form, L-methylfolate, throughout. We note that the full terminology is L-5-methyl-(6S)-tetrahydrofolate, (L-5-MTHF), and the term L-methylfolate is more common, but they refer to the same molecule. We note that in some referenced publications different abbreviations such as L-5-MTHF have been used. In clinical practice, it is always best to use the L-isomer of methylfolate or of folinic acid (leucovorin) in treatment, as there is no dilution from an equal amount of inactive D-isomer found in racemic preparations.

#### 1.1.1 Steps in the folate pathway

Dietary folates (mainly polyglutamated forms) are deconjugated to monoglutamate forms in the small intestine and absorbed via specific transporters (e.g., proton-coupled folate transporter). Once in the bloodstream, folate is transported to tissues and taken up by cells through reduced folate carriers (RFC) and folate receptors (3, 5).

Inside cells, folate is reduced to dihydrofolate (DHF) and then to tetrahydrofolate (THF) by the enzyme dihydrofolate reductase (DHFR). THF acts as a carrier of one-carbon units in various oxidation states, forming derivatives such as 5,10-methylene-THF, 5-methyl-THF, and 10-formyl-THF (3, 6).

THF derivatives participate in purine and thymidylate (dTMP) synthesis (for DNA/RNA); methionine regeneration from homocysteine (via 5-methyl-THF and methionine synthase); and methylation reactions (via S-adenosylmethionine, SAM) (3).

Folate metabolism occurs in both the cytosol and mitochondria, with specific enzymes and folate forms in each compartment. Folate metabolism is tightly regulated, and cells possess repair mechanisms for damaged folate molecules (6, 7).

#### 1.1.2 Folate deficiency/insufficiency and vascular dysfunction

Folate deficiency or insufficiency leads to impaired DNA synthesis, megaloblastic anemia, elevated homocysteine, and it increases the risk of vascular dysfunction, including atherosclerosis, stroke and degenerative diseases (1, 2). Elevated homocysteine due to low folate impairs endothelial function and increases oxidative stress, a key factor in vascular disease (1, 4).

Supplementation with folic acid or L-methylfolate can improve endothelial function, especially in those with cardiovascular risk or established disease, by increasing nitric oxide (NO) bioavailability and reducing oxidative stress (1, 4, 8). Mandatory folic acid fortification in the USA has reduced rates of neural tube defects and improved population folate status, but concerns remain about unmetabolized folic acid (UMFA) with high supplementation, as it may interfere with cellular access or use of L-methylfolate (2, 3).

### 1.2 Vitamins essential for one-carbon metabolism: pyridoxine and cobalamin

Vitamin B6 (Pyridoxine) and B12 (Cobalamin) are both co-factors in homocysteine metabolism, working synergistically with folate to convert homocysteine to methionine (1, 3). Vitamin B6 acts as a coenzyme in the transsulfuration pathway, converting homocysteine to cystathionine and then to cysteine. Adequate B6 prevents hyperhomocysteinemia, thereby supporting endothelial function (1). Additionally, B6 modulates the expression of adhesion molecules and inflammatory cytokines, protecting against endothelial activation and subclinical vascular inflammation (9).

Vitamin B12, in concert with folate, is essential for the remethylation of homocysteine to methionine. Insufficiency exacerbates homocysteine elevation and related vascular threats (10). Vitamin B12, along with folate, is also necessary for myelin synthesis and repair. For this reason, it is a promising treatment for neuropathic pain, as it may promote myelination and increase nerve regeneration (11).

Both vitamins are readily absorbed from dietary and supplemental sources. They both support DNA synthesis and repair (2). Absorption can be impaired in common conditions, including aging and gastrointestinal disorders) (3). Additionally, deficiencies can lead to elevated homocysteine, which creates an increased cardiovascular risk (12).

### 1.3 Public health aspects

#### 1.3.1 Nutritional sufficiency

Population studies underscore the prevalence of suboptimal vitamin intake and its correlation with atherosclerotic disease progression and adverse vascular outcomes. Surveys continue to show many individuals, including in developed nations, fall short of optimal vitamin intake, especially B9, B12, D, and C (13–15). Treatment of these deficiencies could ideally be addressed with cardiovascular-healthy diets (16), and supplementation may be considered in cases of diminished absorption capacity or a genetic or immune condition limiting the bioavailability of these vitamins.

#### 1.3.2 Genetic polymorphisms

Polymorphisms in genes encoding enzymes for folate and homocysteine metabolism such as Methylenetetrahydrofolate Reductase (MTHFR) increase the need for bioavailable forms of folate (such as L-methylfolate or a drug product containing levoleucovorin). These gene variants may exacerbate deficiencies and vascular risk. In these cases, direct use of L-methylfolate (or levoleucovorin) is beneficial (2, 17).

The existence of polymorphisms often raises concerns as to whether there may be variations in efficacy or in negative effects of treatment with a replacement for folate. Because leucovorin has been used in cancer therapy for many decades, including with young children, there is ample evidence that even with much higher doses than discussed here, the administration of leucovorin rescue (referred to as a rescue because it is rescuing the children from the cancer medication methotrexate, which depletes vitamin B9) was not found to have a negative impact, and the positive effect was present for all ethnic groups (18).

#### 1.3.3 Supplementation and disease prevention

Mandatory folic acid fortification has lowered neural tube defect rates. This supplementation must be balanced against issues in those unable to efficiently convert folic acid to its bioactive form. There is increasing evidence that non-deficient individuals and those with gene variants may be negatively impacted by folic acid supplementation (8, 13).

Vitamins are indispensable for healthy development through their diverse roles in cellular proliferation, methylation, antioxidant defense, and endothelial and neuronal function. Timely identification and correction of deficiencies or insufficiencies, targeted supplementation in at-risk individuals, and a foundation of nutrient-rich dietary habits are all strategies with significant potential to support healthy development across the life course, beginning before conception. The remainder of this review focuses on folate (vitamin B9) and vitamin B12, given their particular relevance to neurodevelopmental outcomes.

## 2 Folate sources, absorption and bioavailability

Vitamin B9, commonly known as folate, is an essential water-soluble B vitamin required for DNA synthesis, amino acid metabolism, and key methylation reactions.

Folate exists in both natural forms found in many food products, and as a synthetic compound, folic acid, used in supplementation and food fortification. The natural forms are reduced in form and folic acid is oxidized, making it stable for long periods of time. Understanding natural and synthetic sources, comparative bioavailability, and the impact of genetic variation on folate metabolism is critical for optimizing health and preventing deficiency-related conditions (2, 15, 19, 20).

### 2.1 Folate from foods

Folate occurs naturally in several foods. Food products with the highest folate concentrations are shown in Table 1. Among these, spinach, liver, asparagus, and Brussels sprouts are particularly folate-rich (17, 20). The dominant forms of folate in foods are polyglutamated tetrahydrofolates (such as L-methylfolate), which are critical for metabolic functions. The glutamate residues are removed in the gut to provide the active methylfolate used by cells (21).

**Table 1:** Key food sources of folate.

Food product	Example foods
Dark green leafy vegetables	Spinach, kale, collard greens, and romaine lettuce
Legumes	Beans, lentils, peas
Fruits	Oranges, avocados, bananas, and melons
Nuts and seeds	Peanuts, sunflower seeds
Other vegetables	Asparagus, Brussels sprouts, broccoli, beets
Animal products	Liver and eggs (liver being especially high)
Whole grains	Fortified and unfortified whole grain products

Sources (2, 15, 17, 20).

Folic acid is mono-glutamate, and is fully oxidized and stable. It is chemically distinct from natural food folate in that it does not require deconjugation or release from plant matrices. Fortified foods and supplements provide reliable, high bioavailability forms of folic acid, compensating for losses from natural sources and helping reduce deficiency rates (20, 22, 23). Folic acid is present in fortified grains and dietary supplements (Table 2).

**Table 2:** Effect of maternal folate deficiency as contributors to Autism risk.

Mechanism	Effect of folate deficiency	Link to autism risk
DNA synthesis and repair	Impaired neuron development	Structural/functional brain defects
Homocysteine metabolism	Homocysteine accumulation, neurotoxicity	Disrupted neurodevelopment
DNA methylation (epigenetics)	Hypomethylation, gene dysregulation	Aberrant gene expression in brain
Neurotransmitter synthesis	Imbalanced serotonin/dopamine/norepinephrine	ASD-related behavioral changes
Oxidative stress/inflammation	Increased neuronal damage	Neurodevelopmental risk

Another form of folate is found in medical foods. These are products that contain L-methylfolate (L-5-MTHF), a bioactive form used in certain clinical situations. Leucovorin/levoleucovorin, a bioactive form used in cancer therapy to replace folate depleted by chemotherapy and used as a treatment to alleviate communication symptoms in autism (24), are classified as a drug product. Both are available in the L-form, and in the past they were often available in a racemic mix of the L- and D- isomers. In the racemic case, 50% is the usable L-form, so the dosages needed are twice that of the L-isomers (Table 3).

**Table 3:** Factors in maternal folate intake and related offspring outcomes.

Factor	Timing/context	Outcome/association	Supporting evidence
Maternal leucovorin in women with FRAA	Periconceptional (before and just after conception), through gestation	Both children neurotypical at 3 years; presumed reduction in ASD risk in pregnancy	(65)
Maternal folic acid supplementation	Periconceptional (before and just after conception), 1st trimester	ASD risk decreased by 50% or more	(52, 62–64)
Maternal folic acid supplementation	No supplementation during pregnancy	Potential increased ASD risk; fetal brain may be sensitive to excess micronutrients	(63)
Maternal plasma/whole blood folate concentration	Early pregnancy	Mixed findings; some studies show no direct association with autistic traits	(62)
Maternal plasma folate and B12 (very high levels)	At delivery, 3rd trimester	Potential increased ASD risk; fetal brain may be sensitive to excess micronutrients	(62)
Maternal MTHFR gene variant (C677T)	With low folic acid intake	Stronger association between low maternal folate and increased ASD risk	(62, 64)
Prenatal multivitamin use (with folic acid)	Preconception and 1st month of pregnancy	Reduced ASD diagnosis and symptom severity in children	(52, 62)
Lack of prenatal vitamin/folic acid use	Preconception and 1st month of pregnancy	Higher risk of ASD and more severe symptoms	(52, 62)

### 2.2 Bioavailability and absorption

The bioavailability of naturally occurring folate is inherently variable and generally limited, compared to synthetic folic acid. Typical estimates are that natural food folate is about 50–60% as bioavailable as folic acid consumed with a meal (3, 19, 25). However, there is a limit to the amount of folic acid that can be converted each day, with excess consumption over about 400 ug remaining in the blood as unmetabolized folic acid (UMFA) (2, 3). Additionally, it is estimated that about 10% of the population cannot absorb folic acid across the blood–brain barrier, limiting the utility of this supplement (26).

Three factors affect natural folate bioavailability. These are that folate can be tightly bound within plant cell structures; that natural folate is polyglutamated, which requires enzymatic deconjugation before absorption; and that folate is susceptible to heat, light, and oxidation, so losses can occur during cooking and storage (3, 19, 25).

Emerging research suggests that, depending on the food and preparation, bioavailability may range from roughly 44 to 80%, with a median estimate around 65% as compared to synthetic folic acid (19, 25, 27). Labelling of folate in food products includes indication of the dietary folate equivalent (DFE) which relates that 1 DFE corresponds to 0.6 mg of folic acid or L-methylfolate. This bioavailability variation, along with incomplete liberation from the food matrix and sensitivity to food processing, is a key reason public health campaigns emphasize folic acid fortification, and why this fortification has successfully raised population folate status and reduced the incidence of neural tube defects (3, 20). And it is this same success of folic acid that gives rise to the increase in UMFA, particularly in individuals with larger consumption of the fortified grain products. There are estimates that while the average intake from fortified foods is about 200 ug, individuals may diverge from this by a factor of 5 greater or lesser.

### 2.3 Genetic influences on folate metabolism

Several common genetic polymorphisms influence folate absorption, metabolism, and the body’s methylation capacity.

MTHFR (Methylenetetrahydrofolate Reductase) gene *C677T* and *A1298C* polymorphisms affect the conversion of folic acid and food folates to the bioactive methylfolate form. This impacts homocysteine levels and increases susceptibility to neural tube defects and to ASD in certain populations (28–31). Individuals with these variants may have higher homocysteine levels and may benefit from supplementation with L-methylfolate rather than folic acid (32–34).

*MTR*, *MTRR*, *FOLH1*, *RFC1*, and *SHMT* genes. These variants can influence absorption, tissue distribution, and utilization of folate, with downstream effects on DNA methylation and health outcomes (28–31).

DHFR (dihydrofolate reductase). Variability in DHFR activity can affect the reduction of folic acid to tetrahydrofolate, influencing how efficiently synthetic folic acid is utilized. Excess folic acid may accumulate in individuals with low DHFR activity (35).

### 2.4 Folate transport into the brain

Folate must cross the blood–brain barrier (BBB) to support neurological function. This process is mediated by at least two specific transport proteins. Folate receptor alpha (FRα) is essential for high-affinity transportation of folate across the choroid plexus into the cerebrospinal fluid and brain tissue. Mutations in the FOLR1 gene can cause cerebral folate deficiency (CFD), leading to neurological symptoms (36).

The reduced folate carrier (RFC, *SLC19A1*) facilitates folate entry into various tissues, including the brain, but because it does not use active transport, it can at best replicate plasma folate concentrations into the brain. Cerebral folate levels are typically 2–5 times greater in brain than blood, with youngest children having highest (5X) cerebral folate, and the multiplier diminishes through childhood/adolescence to 2X. Genetic variations in the RFC gene can influence folate delivery to the central nervous system. Recent research also implicates the vitamin D receptor (VDR) in the regulation of folate transport across the BBB, especially in certain neurological diseases (37, 38).

Disruption of these transport mechanisms, whether by genetic mutations, autoantibodies, or disease, can result in cerebral folate deficiency, a low brain folate level, even when blood folate status appears normal. Treatment with leucovorin/levoleucovorin can sometimes bypass these defects and restore brain folate levels (38).

These genetic factors help explain differences between individuals in folate requirements, the varied responses to supplementation, and the risk for conditions linked to both plasma folate deficiency and cerebral folate deficiency, including birth defects, cardiovascular disease and autism spectrum disorder (28–31).

Research continues to elucidate the interaction between genetic variation, environmental exposures, and folate-dependent pathways, pointing to a future with more personalized recommendations on folate intake (39).

## 3 Neurodevelopmental disorders: autism Spectrum disorder

### 3.1 Folate, early brain development, and ASD risk: overview

From a public health perspective, folate status is shaped by dietary intake, supplement use, food fortification, and access to prenatal nutrition counseling. Deficiency during the first month of gestation is a well-established cause of neural tube defects, but folate may also affect broader neurodevelopmental outcomes beyond structural birth defects (40). Experimental studies suggest that inadequate folate during gestation can impair neuronal proliferation, migration, synaptogenesis, and brain organization, raising the possibility that early nutritional insufficiency may contribute to later neurobehavioral differences, including features associated with ASD (41).

Epidemiological evidence supports the relevance of this issue at the population level. Low maternal folate status and insufficient periconceptional folic acid supplementation have been associated with increased ASD risk in offspring, whereas maternal folic acid use around conception has been linked to lower risk in several cohorts (42). Meta-analytic findings have similarly suggested a protective association between maternal folic acid supplementation and ASD risk (43). Although observational studies cannot prove causality, the consistency of these findings across populations suggests that ensuring adequate folate intake before and during pregnancy may represent a practical and potentially modifiable strategy for supporting healthy neurodevelopment (44).

The fetal period is a time of intense susceptibility to folate deficiency. Neural tube closure occurs very early in pregnancy, often before pregnancy is recognized, which is why periconceptional folate intake is so critical. Adequate folate availability during this period is essential for neural tube formation, neuronal proliferation, migration, and synaptogenesis. These same processes later contribute to the organization of functional neural circuits.

ASD is a heterogeneous neurodevelopmental condition with complex and multifactorial origins. Folate is not viewed as a sole cause or cure, but it has attracted significant attention because of its potential role in fetal brain development and epigenetic regulation. Epidemiologic studies have reported associations between periconceptional folic acid use and lower ASD risk in offspring, while inadequate maternal folate status has been linked in some studies to higher risk (41, 45, 46).

These findings do not establish causality, but they are consistent with what is known about folate biology. Because folate supports DNA synthesis and methylation during critical windows of neurodevelopment, insufficient folate could plausibly alter neurodevelopmental trajectories. In this way, folate is relevant not only to structural development but also to later behavioral and cognitive outcomes.

Mechanistic studies strengthen this rationale. Altered DNA methylation patterns have been reported in ASD, including changes in genes involved in synaptic signaling, neuronal connectivity, and neurodevelopment (47). Folate deficiency may also increase homocysteine, which may contribute to oxidative stress and impair developmental signaling pathways (48, 49). Together, these findings support the view that folate-dependent processes may contribute to ASD risk or phenotype through converging metabolic and epigenetic pathways.

### 3.2 Folate role in brain formation during pregnancy

Folate is vital for the closure of the neural tube during embryogenesis, which occurs within the first month of pregnancy. Inadequate maternal folate intake is a well-known risk factor for neural tube defects such as spina bifida and anencephaly. Beyond neural tube defects, folate is crucial for early brain development, influencing neuronal proliferation, migration, and synaptogenesis (41, 50).

Rodent models have demonstrated that maternal folate deficiency leads to impaired neurogenesis, abnormal brain structure, and behavioral changes reminiscent of ASD, such as reduced social interaction and increased repetitive behaviors (51).

Epidemiological studies have linked low maternal folate levels or lack of periconceptional folic acid supplementation to increased ASD risk in offspring. A large Norwegian cohort study found that maternal use of folic acid supplements around conception was associated with a lower risk of autistic disorder in children (52). Similarly, a meta-analysis concluded that maternal folic acid supplementation significantly reduced the risk of ASD (53).

Folate is a key donor of methyl groups for DNA methylation, an epigenetic mechanism that regulates gene expression. Disruptions in methylation pathways have been implicated in ASD. Children with ASD often exhibit abnormal DNA methylation patterns and altered expression of genes involved in synaptic function and neurodevelopment (54). Low folate increases homocysteine levels, which is neurotoxic and can impair neuronal migration, synapse formation, and myelination. Elevated homocysteine during gestation has been associated with increased risk of neurodevelopmental disorders in offspring.

Folate supplementation may help correct these epigenetic disruptions. For instance, animal studies show that maternal folate supplementation can reverse methylation abnormalities and behavioral deficits in offspring exposed to environmental risk factors for ASD (51).

Maternal folate deficiency during early brain development increases autism risk through multiple mechanisms: impaired DNA synthesis and repair, epigenetic dysregulation, and elevated neurotoxic homocysteine. Both animal and human studies support the protective effect of adequate maternal folate against ASD. Ensuring sufficient maternal folate intake before and during early pregnancy is a key public health strategy to reduce the risk of autism and optimize neurodevelopmental outcomes.

Recent clinical trials have investigated the effects of high-dose leucovorin in ASD children, particularly those with mitochondrial dysfunction or folate receptor autoantibodies (55). Multiple trials in which ASD children received high-dose leucovorin (2 mg/kg/day, up to 50 mg/day) for 12 weeks have been conducted. In each, the treatment group showed significant improvements in verbal communication, especially in children with folate receptor autoantibodies (24, 49).

### 3.3 Maternal folate deficiency and autism risk

Decreased access to folate during gestation can be due to low maternal folate levels, genetic variants, or autoimmune disorders (39, 56). Table 4 summarizes the impact of maternal folate deficiency and its link to autism. Insufficient folate conveys multiple risks for neurodevelopmental disorders.

**Table 4:** Randomized and placebo-controlled trials of leucovorin (folinic acid) in children with autism spectrum disorder.

Study (Ref.)	Design	Population	Dose and duration	Key outcome
Frye et al. (24)	Double-blind, placebo-controlled RCT	Children with ASD and language impairment, with or without FRAA	Leucovorin ~2 mg/kg/day, up to 50 mg/day, 12 weeks	Significant improvement in verbal communication, greatest in FRAA-positive children
Renard et al. (EFFET trial) (49)	Placebo-controlled RCT	Children with ASD	Folinic acid, 12-week course	Improved Autism Diagnostic Observation Schedule (ADOS) scores versus placebo
Zhang et al. (71)	Open-label/controlled study with genetic stratification	Children with ASD, stratified by folate-metabolism gene polymorphisms	High-dose folinic acid	Safety and efficacy demonstrated; benefit varied by folate-metabolism genotype
Batebi et al. (72)	Double-blind, placebo-controlled RCT	Children with ASD and inappropriate speech	Folinic acid as adjunctive therapy, 12 weeks	Significant improvement in inappropriate speech versus placebo

FRAA, Folate receptor alpha autoantibodies; ASD: autism spectrum disorder.

#### 3.3.1 Impaired DNA synthesis and neuronal repair

Folate is essential for DNA synthesis and repair, especially during periods of rapid cell division in early embryonic brain development. Deficiency leads to DNA instability and impaired neuronal repair, potentially causing abnormal brain structure and function, key features implicated in autism spectrum disorder (ASD) (57).

#### 3.3.2 Homocysteine accumulation and neurotoxicity

Folate regulates homocysteine metabolism. Without adequate folate, homocysteine levels rise. Elevated homocysteine is neurotoxic. It can disrupt neuronal migration, synapse formation, and myelination, all critical for normal brain development. High maternal homocysteine is associated with increased ASD risk in offspring (58).

#### 3.3.3 Disrupted methylation and epigenetic regulation

Folate provides methyl groups for DNA methylation, a key epigenetic process that controls gene expression during neurodevelopment. Deficiency results in hypomethylation, leading to abnormal activation or silencing of genes crucial for brain development, synaptic function, and neuronal connectivity. Epigenetic dysregulation is a recognized mechanism in ASD etiology (57).

#### 3.3.4 Altered neurotransmitter synthesis

Folate is involved in the synthesis of neurotransmitters such as serotonin, dopamine, and norepinephrine. Deficiency may lead to imbalances in these neurotransmitters, which are often observed in individuals with ASD (59).

#### 3.3.5 Oxidative stress and inflammation

Low folate levels increase oxidative stress and susceptibility to neuroinflammation. Both oxidative stress and chronic inflammation are implicated in the pathogenesis of ASD (40, 60). A valuable biomarker that has systematically indicated children that will respond positively to leucovorin treatment is the presence of folate receptor autoantibody (FRAA) in blood plasma. FRAA is produced by the immune system in response to a dietary product (61). In multiple clinical trials, ASD children with FRAA showed improvement in social communication symptoms when treated with daily leucovorin (up to 2 mg/kg body weight) (24, 49). Larger scale research-controlled trials are needed to determine if this can become standard practice of care, and planning for such a trial is underway.

### 3.4 Critical periods for folate and autism

Research consistently identifies early pregnancy, particularly the periconceptional period (just before and shortly after conception) and the first trimester, as the most critical window for folate intake to reduce autism risk in offspring. Multiple large cohort and case–control studies demonstrate that folic acid supplementation starting before conception and continuing through early pregnancy is associated with a significantly lower risk of autism spectrum disorder (ASD) in children (52, 62–64).

The Norwegian Mother and Child Cohort Study found a 40–45% reduction in autistic disorder risk among women who took folic acid supplements from 4 weeks before to 8 weeks after conception (62). Similarly, a meta-analysis of published studies where folic acid was supplemented during pregnancy (but not prior) found a significant reduction in risk of ASD (53).

When leucovorin was supplemented (at 7.5 mg/day) periconceptionally and throughout gestation in two women expressing FRAA, and with prior ASD births, the children were neurotypical at 3 years of age (65). In a follow-on clinical trial of 210 women planning pregnancy, FRAA was evaluated, and all those who were positive for FRAA were recruited into two arms of the trial: standard folic acid supplementation through the pregnancy, or leucovorin supplementation. When the children were tested for ASD at 30 months, 62% of the folic acid arm children were autistic, while only 10% of the leucovorin arm children were autistic (66).

This indicates a more universal treatment for periconception and gestation of mothers with elevated risk may be leucovorin rather than folic acid.

The first month of pregnancy, when the neural tube forms and closes, is especially sensitive to folate status (58). Supplementation during this period appears to be crucial for both preventing neural tube defects and reducing ASD risk.

Continuous folic acid supplementation from the periconceptional through the prenatal period may offer the greatest protective effect to reduce ASD risk (62, 63, 65, 66). Women who missed supplementation during both periods had the highest risk of having a child with ASD (62, 63).

Some evidence suggests a U-shaped relationship: both low and excessively high maternal folate levels may increase ASD risk, while moderate, recommended intake is optimal (62). This may be unique to folic acid supplementation, as a study with 7.5 mg/day of leucovorin did not exhibit any high folate risk (65, 66).

#### 3.4.1 Insights from folate supplementation in reducing risk of autism

An important critical period for folate intake to prevent autism is from at least 1 month prior to conception through the first trimester of pregnancy. Supplementation during this window is strongly associated with a reduced risk of ASD in offspring (52, 62–64, 66).

Overall observations are:

- Begin Supplementation Before Conception: Start at least 1 month before trying to conceive and continue through the first trimester of pregnancy (52, 62–64, 66).
- Maintain Adequate Intake: 400 micrograms (mcg) of folic acid daily is the widely recommended dose for women planning pregnancy and during early gestation (58, 63).
- Continue Through Early Pregnancy: Ensure consistent intake through the first 2–3 months of pregnancy, the period of neural tube and early brain development (58, 63).

Maternal folate is crucial for neurodevelopment during critical pregnancy windows due to its central role in several fundamental biological processes required for healthy brain formation and function in the fetus. We recommend trials to confirm and expand these findings.

#### 3.4.2 DNA synthesis and cellular proliferation

Folate is essential for DNA synthesis and repair, supporting the rapid cell division and growth that occur during early embryonic development. This is especially important for neural stem cell proliferation and differentiation, which lay the foundation for the brain’s structure and complexity (41, 67, 68). The roles of folate in early development include:

- Neural Tube Closure. During the first month of pregnancy, folate is critical for the closure of the neural tube—a process that, if disrupted, results in severe neural tube defects such as spina bifida and anencephaly. This is why periconceptional folic acid supplementation is universally recommended to women planning pregnancy (41, 67–69).
- Epigenetic Regulation and Gene Expression. Folate acts as a methyl donor in one-carbon metabolism, which is required for DNA methylation. Proper methylation regulates gene expression patterns necessary for normal brain development. Disrupted methylation due to folate deficiency can lead to long-lasting neurodevelopmental and cognitive changes in offspring (41, 67, 68).
- Neurotransmitter and Phospholipid Synthesis. Folate is involved in the synthesis of key neurotransmitters (serotonin, dopamine, norepinephrine, acetylcholine) and phospholipids, both of which are vital for neuronal signaling, connectivity, and myelination (41, 67, 68).
- Prevention of Cell Death (Apoptosis). Adequate maternal folate reduces apoptosis (programmed cell death) in developing brain regions, ensuring proper formation of neural circuits (68).
- Maintenance of Healthy Homocysteine Levels. Folate helps maintain low homocysteine concentrations. Elevated homocysteine is neurotoxic and has been linked to impaired neurodevelopment (41, 67).

Human studies, while sometimes inconsistent due to methodological differences, generally support that adequate maternal folate, especially during the periconceptional period and first trimester, is associated with better neurodevelopmental and cognitive outcomes in children (41, 67, 68, 70). This is summarized in Table 5.

**Table 5:** Summary of the pilot randomized trial of calcium folinate versus folic acid in FRAA-positive pregnancies (66).

Parameter	Value
Women screened for FRAA (preconception)	210
FRAA-positive (% of screened)	36 (17.1%)
FRAA-positive women who conceived and were randomized	29
Completed follow-up	18
Calcium folinate arm (n)	10
Folic acid arm (n)	8
ASD incidence, calcium folinate arm	1/10 (10%)
ASD incidence, folic acid arm	5/8 (62.5%)
Treatment duration	From pregnancy confirmation until delivery
Notable confounder	One ASD case in the calcium folinate arm carried a pathogenic SHANK2 variant

FRAA, Folate receptor alpha autoantibodies; ASD, Autism spectrum disorder. Sample sizes are small and findings should be interpreted as preliminary.

Maternal folate is vital during critical pregnancy windows because it underpins DNA synthesis, gene regulation, neurotransmitter production, and neural tube closure, all of which are foundational for healthy fetal brain development (41, 67).

#### 3.4.3 Insights from folate supplementation in reducing risk of autism

- Periconceptional and first trimester folate intake are most strongly associated with reduced ASD risk and better neurodevelopmental outcomes (52, 62–66)
- Very high maternal folate/B12 levels late in pregnancy may be associated with increased ASD risk, suggesting moderation is important (62)
- Genetic factors (e.g., MTHFR polymorphisms) can modify the protective effect of folate (62, 64)
- Plasma/whole blood folate levels alone are less predictive than supplement use, possibly due to timing and individual metabolism (62)
- Use of leucovorin during periconceptional period and throughout gestation may mitigate the risk associated with gene variants and maternal FRAA (65, 66)

Evidence from epidemiologic studies suggests that folate exposure during the periconceptional period and early pregnancy may be particularly important for ASD risk (40, 57, 65–68). Large cohort and case–control studies have reported that folic acid supplementation started before conception and continued through early gestation is associated with a lower risk of ASD in offspring (40, 57, 65–67). In the Norwegian Mother and Child Cohort Study, maternal folic acid use from 4 weeks before to 8 weeks after conception was associated with a substantially lower risk of autistic disorder in children. Meta-analytic findings have also supported an inverse association between maternal folic acid supplementation and ASD risk. Together, these studies suggest that adequate folate intake during early pregnancy may support favorable neurodevelopment and may represent a modifiable exposure of public health importance (40, 57, 65–68).

The first month of pregnancy appears to be a particularly sensitive period, because neural tube closure and early neurodevelopment occur during this time. This timing reinforces the importance of preconception counseling and early prenatal nutrition guidance, since folate needs are greatest before pregnancy recognition in many cases. Evidence also suggests that uninterrupted supplementation across the periconceptional and first-trimester periods may offer the greatest benefit, highlighting the role of timely and sustained folate intake in pregnancy care.

Interest has also expanded beyond prevention to the potential role of folate-related treatment after birth. Clinical trials of folinic acid in ASD children have reported improvements in some symptom domains, particularly language and communication-related outcomes in certain subgroups (27, 51, 58). Although these findings remain preliminary, they suggest that folate metabolism may remain relevant beyond gestation and may have implications for targeted nutritional intervention in selected children with ASD.

Taken together, the epidemiologic, mechanistic, and early clinical trial evidence supports continued investigation of folate across the life course, from preconception and pregnancy through childhood. For nutrition professionals, this literature underscores the importance of adequate folate intake before and during pregnancy, as well as the need to clarify which children, if any, may benefit from folate-related therapeutic strategies later in development.

### 3.5 Nutritional and clinical practice implications

For nutritionists and dietitians, folate is important across the life course, but especially in the reproductive years. Preconception nutrition counseling is essential because folate-dependent developmental events occur before pregnancy is often clinically recognized. Standard public health guidance emphasizes folic acid supplementation before conception and during early pregnancy, primarily to prevent neural tube defects (65, 67, 68). However, the growing literature on folate and ASD suggests that folate status may also have relevance for broader neurodevelopmental outcomes (69–72).

This has increased interest in whether different folate forms may matter in specific populations. Folic acid remains the mainstay of public health fortification and supplementation, but some individuals may benefit from reduced folate forms such as L-methylfolate or folinic acid, especially if folate transport or conversion is impaired. These options are not yet substitutes for population-level prevention strategies, but they are increasingly discussed in precision nutrition and therapeutic contexts (69).

Nutrition professionals should also consider that folate status cannot be interpreted in isolation. Vitamin B12 status, homocysteine levels, genetic variation, maternal immune factors, and overall dietary quality all influence folate metabolism and its biological effects. In practice, this means folate counseling should be contextual rather than one-size-fits-all.

From a dietetics perspective, folate assessment should include dietary intake, supplement use, timing relative to conception, and risk factors for impaired folate metabolism. Women planning pregnancy should be counseled that folate needs begin before conception, not after a positive pregnancy test. This is especially important because many neural developmental processes occur in the earliest weeks of gestation.

In families affected by ASD or other developmental disorders, folate status may be one component of a broader nutritional and metabolic assessment. Although folate is not a treatment for ASD, its role in neurodevelopment makes it an important nutrient to evaluate when discussing maternal and child nutrition. Dietitians may also need to interpret folate findings in relation to B12 status, dietary patterns, and possible use of folate-containing supplements or fortified foods.

The practical message is that folate should be viewed as both a preventive nutrient and a developmental regulator. Its importance lies not only in preventing a known deficiency syndrome, but also in supporting the biochemical environment required for healthy fetal brain development.

A second reduced folate form, folinic acid, also called leucovorin, is distinct from folic acid. It does not require the same initial reduction step and can enter folate metabolism downstream of folic acid. In clinical contexts, this distinction matters because some individuals may have impaired conversion or transport of folate forms. The biologically active L-isomers are the clinically relevant forms; racemic preparations may contain inactive D-isomers that dilute potency.

Folate is a biologically central vitamin with major implications for growth, methylation, and neurodevelopment. Its chemistry, transport, and conversion pathways determine how effectively it supports one-carbon metabolism and developmental programming (7, 8, 73). For nutritionists, folate is therefore more than a routine prenatal nutrient; it is a key determinant of reproductive and developmental health.

Its relevance to ASD and other developmental disorders reflects the intersection of nutrition, epigenetics, and early brain development (74, 75). While more research is needed to clarify the roles of folate form, timing, and individual susceptibility, the current evidence supports careful attention to folate across preconception, pregnancy, and early life.

## 4 Detection of folate deficiency in children and treatment

### 4.1 Detection: folate receptor alpha autoantibodies and cerebral folate deficiency

Giorlandino et al., 2026, extended this line of research by testing whether correcting impaired folate transport during pregnancy could influence early neurodevelopmental outcomes in offspring (66). Their work is mechanistically grounded in FRAA, which can interfere with placental and blood–brain barrier transport of folate and reduce delivery of 5-methyltetrahydrofolate to the fetal brain despite apparently adequate maternal folate status (66, 69). This pathway is especially relevant because FRAA have been reported more frequently in ASD children and in mothers of children affected by ASD, suggesting that disrupted folate transport may define a biologically distinct subgroup within the broader ASD spectrum (26, 58).

### 4.2 Leucovorin clinical trials in children with ASD

Leucovorin (5-formyl-tetrahydrofolate, folinic acid) is a bioactive form of folate that bypasses certain metabolic blocks, including those caused by FRAA or mitochondrial dysfunction, both of which are more prevalent in many children presenting with autism spectrum disorder. FRAA are present in approximately 70% of ASD children (24). Mitochondrial dysfunction is estimated to occur in up to 5% of children with ASD, and it can impair folate transport into the brain, potentially worsening neurodevelopmental symptoms (55).

Estimates of FRAA prevalence in ASD vary across studies, ranging from roughly 40–70%, depending on the assay platform, the antibody isotype measured (blocking versus binding autoantibodies), and the reference population used for comparison. FRAA testing is not yet standardized or widely available outside of specialized research or commercial laboratories, and there is no consensus threshold defining a clinically meaningful positive result. These factors should be considered when interpreting reported prevalence figures and when evaluating FRAA as a candidate biomarker for clinical use.

Clinical trials using leucovorin have been completed in multiple countries, with similar findings in all (24, 49, 71, 72). All but one of the trials were double-blind, placebo controlled, with children under 15. The intervention used was leucovorin at 2 mg/kg/day (up to 50 mg/day for 12 weeks). All found significant improvement in verbal communication in a majority (but not all) of children presenting with FRAA, and little to no effect in children testing negative for the antibody. Leucovorin was well-tolerated, with mild side effects of irritability and insomnia being rare and manageable.

The findings indicate that leucovorin can lead to clinically meaningful improvements in social communication, especially language and communication, in children with mitochondrial dysfunction or cerebral folate deficiency (24, 49, 71, 72).

Benefits are most pronounced in subgroups with biomarkers indicating impaired folate transport or metabolism, as measured by presence of FRAA.

Leucovorin is effective due to three features:

- Bypasses Blocked Folate Transport. Leucovorin is rapidly metabolized to L-methylfolate, which can cross the blood–brain barrier via alternative transporters, even when folate receptor alpha is blocked by autoantibodies or the individual has a genetic polymorphism reducing active transport.
- Supports Mitochondrial Function. Leucovorin may enhance mitochondrial energy production, reduce oxidative stress, and improve neuronal metabolism.
- Restores Methylation. As a methyl donor, it supports DNA methylation and neurotransmitter synthesis, processes often disrupted in ASD.

The limitations to leucovorin treatment are that not all children respond. The most significant benefits are seen in children with FRAA and/or with mitochondrial dysfunction. Because most studies are short-term (12–24 weeks), long-term efficacy and safety requires further research.

These trials also have methodological limitations that temper interpretation. Sample sizes were modest (typically 20–60 participants per arm), and only one of the trials summarized in Table 4 was not double-blind, which may introduce assessment bias in that study. Outcome measures varied across trials (e.g., ADOS scores, verbal communication ratings, clinician-rated speech), limiting direct comparability and precluding formal meta-analysis. Most cohorts were drawn from single centers or single countries, which may limit generalizability across genetic and dietary backgrounds. Finally, FRAA-positive subgroups were often defined *post hoc* or with center-specific assay cutoffs, raising the possibility of selection or measurement bias in subgroup analyses.

High-dose leucovorin is an effective and generally well-tolerated intervention for improving social communication symptoms, particularly language and communication, in ASD children who have mitochondrial dysfunction or FRAA. The strongest evidence comes from randomized controlled trials and open-label studies. Benefits are most pronounced in biomarker-positive subgroups. Further research is needed to optimize dosing, duration, and identify all responsive phenotypes (24, 49, 71, 72).

## 5 Detection of folate deficiency risk in pregnancy and treatment

### 5.1 Detection: maternal folate receptor alpha autoantibody screening

In their earlier case observations, mothers with folate receptor alpha autoantibodies who used calcium folinate throughout pregnancy gave birth to children without developmental disabilities at age 3 years (65). The 2026 pilot randomized trial added further evidence by comparing calcium folinate with folic acid in FRAA-positive pregnancies. Offspring in the calcium folinate group showed lower ASD incidence, reduced symptom severity, and higher cognitive scores than those in the folic acid group, although the small sample size limits the strength of inference (66). These findings are consistent with postnatal studies in which leucovorin has been associated with improvements in communication and irritability in children with cerebral folate deficiency or ASD, and with experimental data showing that maternal folate receptor antibody exposure can induce ASD-like behavioral changes that leucovorin may prevent (26, 27).

Taken together, these studies suggest that the form of folate may be clinically important when folate transport is impaired. They also support a more precision-oriented approach to folate nutrition in pregnancy, in which maternal antibody status, folate metabolism, and biological availability are considered alongside total intake. For nutrition and public health practice, this literature raises an important question: whether standard folic acid supplementation is sufficient for all pregnancies, or whether select high-risk groups may benefit from alternative reduced folate forms such as folinic acid, and how to advise parents in considering this option.

### 5.2 Pilot randomized trial: calcium Folinate versus folic acid in FRAA-positive pregnancies

**Figure 2:**
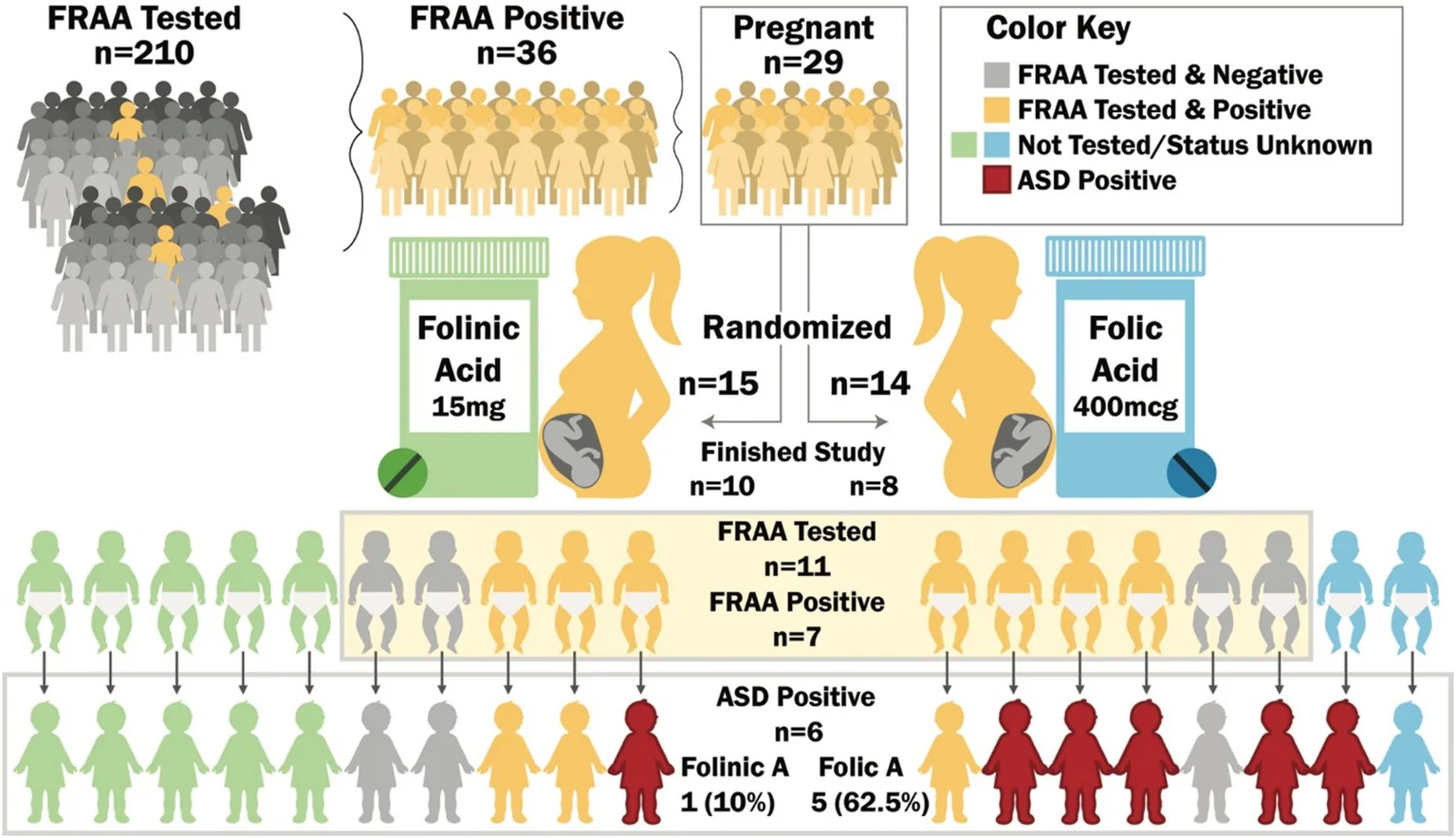
Visual depiction of trial. Half the recruited women received folic acid and half received leucovorin. Children were assessed for autism by 30 months. Of the leucovorin arm, one child was diagnosed as ASD for a 10% prevalence; of the folic acid arm, 5 children were diagnosed as ASD for a 62.5% prevalence (66). Image adapted from Ayoub (82), licensed under CC BY 4.0.

In this context, folinic acid has emerged as a potentially important alternative form of folate in selected pregnancies at elevated risk for adverse neurodevelopmental outcomes. Recent clinical and observational studies suggest that calcium folinate may be more effective than folic acid in some high-risk settings, particularly among women with folate receptor alpha autoantibodies or folate metabolism polymorphisms (46, 65, 66). In a pilot randomized trial, prenatal calcium folinate supplementation was associated with lower ASD incidence, reduced symptom severity, and higher cognitive scores in offspring compared with folic acid, although the sample size was small and the findings should be interpreted cautiously (66). Figure 2 depicts a schematic summary of this trial and its findings. These results are consistent with earlier observational reports in which folinic acid supplementation during subsequent pregnancies was associated with favorable developmental outcomes in later-born children (66).

These findings are clinically relevant because they suggest that the form of folate may matter, not only the dose or timing. While folic acid remains the standard supplement used in population-level prevention strategies, folinic acid may have a role in specific subgroups in whom folate transport or metabolism is impaired. At the same time, evidence from preclinical studies indicates that excessive prenatal folic acid exposure may influence methylation and gene expression in the developing brain, raising the possibility that both too little and too much folic acid could be relevant in susceptible populations (76). This reinforces the need for a more individualized approach to folate nutrition in pregnancy rather than a one-size-fits-all model.

As the authors of the pilot study note, this work requires replication in a larger cohort before it can inform clinical guidance. Of the 29 FRAA-positive women randomized, only 18 completed follow-up, and the resulting per-arm samples (n = 10 and n = 8) are too small to rule out chance findings or to adjust for confounders such as maternal age, ethnicity, or co-occurring genetic risk factors (e.g., the SHANK2 variant noted in one calcium folinate–arm case). The trial was also conducted at a single site, and blinding of outcome assessors, should be confirmed. Given the safety profile of leucovorin from its FDA approved use as folate rescue following methotrexate chemotherapy (76), at the lower doses used in the Giorlandino study, the safety profile of leucovorin is promising. Thus, replication of the pilot study in a larger, multi-site, adequately powered trial is a reasonable and important next step to confirm leucovorin may be a good substitute for folic acid in FRAA positive pregnancies, and to ensure leucovorin serves as a useful folate substitute for folic acid in reducing neural tube defects.

From a nutrition and public health perspective, these data support continued attention to the timing, form, and adequacy of folate exposure across the reproductive period. They also highlight the importance of integrating maternal nutritional status, genetic susceptibility, and emerging biomarker data when considering folate-based strategies for prevention. As a whole, the current evidence suggests that folate-related interventions may influence neurodevelopment not only through overall intake but also through differences in biological availability and maternal-fetal metabolism.

In the pilot randomized trial, 210 women planning pregnancy were screened, and 17.1% were found to be folate receptor alpha autoantibody positive, indicating that this potential risk factor is not uncommon in the preconception population. Among the 29 FRAA-positive women who conceived and were randomized in this study, 18 completed follow-up, with 10 receiving calcium folinate and 8 receiving folic acid. Supplementation was initiated after pregnancy confirmation and continued until delivery (66). Although the per-protocol sample was small, the developmental signal was notable: ASD incidence at 24 to 30 months was substantially lower in the calcium folinate group than in the folic acid group, and children in the calcium folinate arm also showed lower ASD symptom severity and higher cognitive scores.

These findings suggest that when folate transport is impaired by maternal autoantibodies, the form of folate treatment may be clinically relevant. The results are consistent with the concept that folinic acid can bypass some transport limitations associated with folate receptor dysfunction, whereas folic acid may be less effective in this subgroup. The observation that one child in the calcium folinate group had a pathogenic SHANK2 variant also underscores that folate-related intervention may modify one pathway of risk without preventing ASD arising from strong genetic causes (66).

From a nutrition and public health perspective, these data support continued investigation of folate form, maternal immune status, and early pregnancy supplementation strategies. They also reinforce the broader point that adequate folate exposure is not simply a matter of total intake, but may depend on biological availability and maternal-fetal transport. In sum, the trial adds to a growing literature suggesting that precision approaches to folate nutrition may be warranted in selected high-risk pregnancies.

### 5.3 Rationale: why Leucovorin may outperform folic acid in at-risk pregnancies

Standard prenatal folic acid recommendations assume intact folate receptor alpha-mediated transport to deliver 5-methyltetrahydrofolate to the placenta and fetal brain. In folate receptor alpha autoantibody-positive pregnancies, however, this transport pathway may be partially blocked or dysfunctional, allowing cerebral folate deficiency to occur despite apparently adequate circulating folate (74, 77). This limits the expected benefit of folic acid in a subgroup where folate delivery to target tissues is impaired.

Leucovorin differs from folic acid in ways that may be clinically meaningful in this context. It can enter cells through alternative transport systems, including the reduced folate carrier and proton-coupled folate transporter, which may help bypass antibody-related impairment of folate receptor alpha. It is also already in the natural, reduced, biologically active form, which may more directly support nucleotide synthesis and methylation reactions required for neurogenesis and synaptogenesis (73, 77, 78). This is in contrast to folic acid, which is synthetic folate in oxidized form, and thus must be converted to the bioactive, reduced form within the body prior to becoming usable. These properties make leucovorin a plausible alternative when receptor-mediated folate transport is compromised.

Preclinical data further suggest that folate form may matter for neurodevelopmental safety as well as efficacy. Some studies have raised concern that high-dose folic acid may be associated with adverse neurodevelopmental effects in certain settings, whereas leucovorin has shown neuroprotective effects without the same concern profile (47). Although these findings may not support replacing folic acid broadly in public health practice, they do underscore that folate compounds are not interchangeable simply because they increase total folate exposure.

These observations support the idea that leucovorin may be a more physiologically appropriate folate source in selected situations, particularly when folate receptor-mediated transport or folate metabolism is impaired. For nutrition and public health practice, the implication is that folate strategy may need to consider not only dose and timing, but also the specific form of folate and the biological context in which it is given.

An additional line of evidence comes from clinical observations in women with infertility and MTHFR polymorphisms, in whom changing the form of folate rather than the dose appeared to improve reproductive outcomes. In a case series, women with infertility who had previously used folic acid were switched to reduced folate formulations, including leucovorin or methylfolate, and subsequently achieved successful conceptions and healthy births after earlier unsuccessful attempts (79). These findings suggest that folate form may be clinically relevant not only for neurodevelopmental risk but also for fertility and early pregnancy success (80).

Taken together with the findings in folate receptor alpha autoantibody-positive pregnancies, this literature supports the idea that standard folic acid supplementation may not be equally effective across all biological contexts. Both immune-mediated impairment of folate transport and genetically mediated differences in folate metabolism may respond more favorably to reduced folate forms. For nutrition and public health practice, this reinforces the need to consider maternal phenotype, folate metabolism, and biological availability when evaluating prenatal folate strategies.

These observations also broaden the translational relevance of folate research. Rather than viewing folic acid and leucovorin as interchangeable sources of the same nutrient, the current evidence suggests that folate formulation may influence both conception and fetal neurodevelopment in selected populations. This supports further investigation of individualized folate approaches in preconception care and early pregnancy, particularly among women with infertility, metabolic variants, or evidence of impaired folate transport.

### 5.4 Risks of excess folic acid during pregnancy

Preclinical evidence suggests that more folic acid is not necessarily better. High-dose folic acid exposure during vulnerable developmental windows has been shown to alter cortical DNA methylation and gene expression networks, indicating that supraphysiologic exposure to synthetic folic acid can influence neurodevelopmental trajectories in ways that may not be benign (47). Clinical observations have also raised concern that folic acid supplementation later in pregnancy may be associated with less favorable child developmental outcomes, including higher behavioral problems and poorer cognitive development in some cohorts (48).

These findings are important because they challenge the assumption that increasing folic acid intake is always protective. They also suggest that folate strategy may need to account for the biological form of the nutrient, not just the total amount consumed. In susceptible subgroups, including women with folate receptor alpha autoantibodies, MTHFR polymorphisms, or other metabolic constraints, high folic acid exposure could create a mismatch between circulating folate and intracellular utilization, potentially contributing to epigenetic perturbations in the developing brain.

Against this backdrop, leucovorin appears to be a mechanistically attractive alternative. As a reduced folate, it can support one-carbon metabolism while bypassing some transport and metabolic bottlenecks that may limit the effectiveness of folic acid. This may help explain why leucovorin has shown promise in prior studies of ASD, folate receptor alpha autoantibody-positive pregnancies, and infertility. Overall, the preclinical and clinical evidence suggests that folate form may be a critical determinant of benefit, particularly in populations with impaired folate handling.

For nutrition and public health practice, these data reinforce the importance of tailoring folate recommendations to biological context. They also support further investigation into whether reduced folate forms may offer advantages over folic acid in selected high-risk pregnancies and other vulnerable populations.

### 5.5 Toward precision folate strategies in pregnancy

The Giorlandino trial raises important translational questions for obstetrics, neurology, and nutrition regarding screening and targeted supplementation. Folate receptor alpha autoantibodies have been reported in a substantial proportion of mothers of ASD children, and they were present in 17.1% of women planning pregnancy in this trial, suggesting that they may represent a feasible biomarker for stratifying neurodevelopmental risk and guiding folate formulation. This 17.1% figure comes from a single cohort screened at one center, and population-level FRAA prevalence in women of reproductive age more broadly remains uncertain. Larger, more diverse population screens, using a harmonized assay and cutoff, will be needed before FRAA prevalence estimates, or FRAA testing itself, can be generalized to guide preconception screening policy.

In folate receptor alpha autoantibody-positive pregnancies, calcium folinate was associated with lower ASD incidence, reduced symptom severity, and improved cognitive outcomes, whereas folic acid did not provide comparable protection (66).

These findings, while preliminary, are hypothesis-generating, supporting larger trials toward developing a broader reconsideration of prenatal folate policy. Current recommendations were developed primarily to prevent neural tube defects and remain centered on folic acid, but they do not account for FRAA, folate metabolism variants such as MTHFR polymorphisms, or the possibility that excess folic acid exposure may have unintended neurodevelopmental effects in susceptible populations. In this context, reduced folates such as leucovorin may deserve further study as an alternative strategy in selected high-risk groups.

For nutrition and public health practice, the key implication is that folate recommendations may need to move beyond a uniform dosing model and toward a more individualized framework that considers immune status, genotype, and folate form. Larger multicenter studies will be needed to confirm whether screening and targeted supplementation can improve developmental outcomes, but the current evidence suggests that precision folate nutrition may have meaningful relevance for preconception and pregnancy care.

Perhaps the most important conceptual advance from this body of evidence is the integration of maternal autoimmunity and genetic variation into prenatal nutrition and ASD prevention frameworks. Folate receptor alpha autoantibody-mediated cerebral folate deficiency links immunology, metabolism, and neurodevelopment, while the infertility and preclinical folic acid data add genetic and epigenetic dimensions to the story. Together, these findings suggest a more complex model of folate biology than is captured by one-size-fits-all folic acid supplementation.

Future research should extend folate receptor alpha autoantibody studies beyond ASD to other neurodevelopmental outcomes, including language delay, learning disorders, and attention-deficit/hyperactivity disorder. Additional work is also needed to harmonize assays, thresholds, and reporting standards so that folate receptor alpha testing can be evaluated more rigorously for clinical use. In parallel, studies should examine the optimal timing, dose, and formulation of reduced folates, including whether preconception initiation offers greater benefit than supplementation begun after pregnancy confirmation.

Thus, the current evidence supports a more individualized approach to folate nutrition in pregnancy, one that considers maternal immune status, genetic background, and folate form. For nutrition and public health practice, the question is no longer simply whether folate matters, but which folate form, for which women, and at what stage of reproduction it may be most effective.

Important limitations across the full body of evidence that must be addressed include the heterogeneity of outcome measures across the pediatric and prenatal literatures, a reliance on a small number of research groups (particularly for the FRAA-pregnancy data), an absence of independent replication of the Giorlandino pilot trial, and a lack of longer term (multiyear) follow-up data for the pediatric leucovorin trials (NB the Giorlandino trial has a follow-up report at 5 years that confirms the ASD and neurotypical diagnoses they found in their study, with this report under journal review).

## 6 Conclusion

Taken together, the available evidence suggests that leucovorin may have relevance at two stages of the life course: as a potential adjunctive intervention in ASD children and as a candidate preventive strategy during pregnancy in selected high-risk families. In ASD children, existing trials support the possibility that leucovorin improves some neurodevelopmental symptoms.

Of particular interest is the hypothesis that leucovorin use during pregnancy could reduce ASD risk in offspring from pregnancies at elevated risk, especially when folate receptor alpha autoantibodies (FRAA) are present in the blood of either parent. Because FRAA may interfere with placental or fetal folate delivery, targeting this pathway offers a biologically plausible strategy for improving fetal folate availability during critical periods of brain development. This approach is especially relevant in families with evidence of folate receptor autoimmunity, where standard practice of using folic acid supplementation may not address the impaired folate transport.

The preventive role of leucovorin in pregnancy remains investigational. Well-designed prospective studies and randomized controlled trials are needed to determine whether maternal leucovorin supplementation can routinely lower ASD risk, identify the pregnancies most likely to benefit, and define whether parental FRAA status should be used to guide clinical decision-making. One benefit is the historic safety profile of leucovorin; after decades of use it is known to be safe at these levels and higher (76), allowing its use to be considered while studies progress. If confirmed, this strategy could expand specific folate-based prevention beyond general prenatal supplementation toward a more targeted approach for higher-risk pregnancies.
